# Stricture of the Œsophagus

**Published:** 1901-11-02

**Authors:** 


### STRICTURE OF THE ŒSOPHAGUS.

When a patient suffering from œsophageal stricture has been treated by gastrostomy the management of the wound and the protection of the surrounding skin are often found to give both patient and surgeon sufficient occupation, and the original mischief, namely, the stricture, is apt to be left to its own devices. It should, however, be remembered that many œsophageal strictures have been rendered permeable by the method of Socin, which consists in letting the patient wash down with his fluid food (when he can swallow fluid food, which is not always the case) a small leaden shot to which is attached a fine silk thread. This shot with its attached thread is then fished out, or washed out from the gastrostomy wound, and larger and larger threads, and finally instruments are pulled through, it being found easy in this manner to dilate many strictures which it has been impossible to deal with by means of bougies. The difficulty in carrying out this manœuvre lies in the fact that the leaden sinker cannot always be fished or washed out of the stomach. To obviate this trouble, however, Henle[1] has made the "sinkers" of iron, and has caught them in the stomach by means of an iron sound converted into a strong magnet by an electric current being passed through a coil around its handle, after the manner of, but on a larger scale than, the electro-magnets used for the extraction of fragments of iron from the interior of the eye. When this instrument is introduced through the gastrostomy wound it soon attracts the "sinkers." On this happening the patient sometimes feels the drag on the thread, or the surgeon may hear the click, and on withdrawing the magnetic sound the iron sinker with its attached thread is brought out of the wound, after which the course is clear for treatment of the stricture by dilation.

[1] Centralblat fur Chirurgie, No. 34.

